# Perioperative changes in inflammatory biomarkers and underlying molecular mechanisms in patients with trigeminal neuralgia undergoing surgical interventions

**DOI:** 10.3389/fneur.2025.1633630

**Published:** 2025-10-10

**Authors:** Cheng-Jie Qiu, Zhi-Ye Cui, Qi Zhang, Si-Jian Pan, Ben-Gen Pei

**Affiliations:** ^1^Department of Neurosurgery, Ruijin Hospital, Shanghai Jiao Tong University School of Medicine, Shanghai, China; ^2^Department of Neurosurgery, Shanghai University of Medicine & Health Sciences Affiliated Zhoupu Hospital, Shanghai, China

**Keywords:** trigeminal neuralgia, inflammation, biomarker, neurosurgery, perioperative change

## Abstract

Trigeminal neuralgia (TN) is a very painful neurological condition with unilateral and electric shock-like pain attacks. The accurate diagnosis of the disease is of extreme importance for the determination of subsequent therapeutic strategies and clinical management. Surgical interventions including peripheral neurectomy, microvascular decompression (MVD), percutaneous balloon compression (PBC) and stereotaxic radiosurgery (SRS) are options for refractory patients. The utilization of proper perioperative biomarkers in serum, CSF and saliva may help in tracking the safety, efficacy and prognosis after surgical treatments. This narrative review aimed to identify potential inflammatory biomarkers that reflected perioperative changes in clinical practice and explored contributions of inflammation to pathogenesis of the disease. A total of 142 records and 95 clinical trials were identified through structured literature search and underwent subsequent selection with inclusion and exclusion criteria. We summarize relevant literature of current clinical and laboratory findings of the alterations in inflammatory biomarkers in patients with TN before and after the surgical interventions to find out biomarkers for clinical use. We then discuss the underlying molecular mechanisms based on the results from animal models for a better understanding of the role of inflammation in TN and future directions for clinical trials and basic research. Pro-inflammatory cytokines and chemokines, such as IL-1β, IL-6, TNF-ɑ reached high levels in serum, CSF or saliva specimens from TN patients, which could be reversed by PBC, but not always by MVD. The elevated preoperative level of TRAIL was reversable by MVD, but the elevated preoperative level of TNF-*β* was not. These alterations in inflammatory biomarkers were modulated by a variety of signaling pathways, including MAPK- or P2X7- associated pathways. Alterations in these inflammatory biomarkers could be indicative to the perioperative status of TN patients and may be used as additional outcome measurements other than pain relief in clinical trials, however, the consistency in such alterations would need to be verified in larger-scaled clinical studies.

## 1 Introduction

Characterized by unilateral, paroxysmal, electric shock-like, pricking and relapsed pain attacks with sudden onset and sudden termination, trigeminal neuralgia (TN) is a debilitating neuropathic facial pain syndrome affecting the sensory distribution of one or more divisions of the fifth cranial nerve—trigeminal nerve, particularly at the innervation area of the three trigeminal nerve branches, when triggered by innocuous stimuli (1). Studies from animal models showed that persistent external stimulation was transmitted from the affected trigeminal nerve branches into the trigeminal ganglion (TG), resulting in the activation of the TG neurons, which is one of the leading reasons for the occurrence and maintenance of hyperalgesia in these patients (2). TN can be classified as (i) classical TN, which has no apparent cause other than neurovascular compression (NVC) of the trigeminal nerve at the root entry zone (REZ) (3); (ii) idiopathic TN, which shows no significant abnormalities assessed by electrophysiological tests or MRI; and (iii) secondary TN, which is caused by underlying diseases such as brain tumor or multiple sclerosis (MS) (4). The REZ is where the trigeminal nerve enters the brainstem and represents a transition zone from peripheral Schwann cell-mediated myelination to central oligodendrocyte-mediated myelination, which makes this area very susceptible to pressure-induced lesions (5). TN overall affects 0.07% (6) to 0.3% (7) of the general population, with a slight female predominance (approximately 1.5 to 2.1 ratio for male to female) (8, 9). Commonly seen above 50 years of age, its general incidence climbed with age (9). Moreover, the incidence of TN in systemic sclerosis and MS is significantly higher than that in general populations (10), and it has also been associated with other inflammatory diseases such as myositis, arthritis and interstitial lung disease (10). Pathophysiologically, compression from the blood vessel focal accounts for the main cause of TN, leading to demyelination in REZ of the primary sensory trigeminal afferents (11). Surprisingly, changes in the trigeminal nerve has been observed not only on the side with NVC, but also contralaterally, suggesting that individuals with TN may have preexisting abnormalities in the white matter with neuroinflammation and oedema (12). The chronic neuroinflammation of the TG is caused by the persistent constriction of blood vessels in abnormal positions, leading to the development of demyelination in TN patients (13). Indeed, a pivotal role of inflammation in the etiology and progression of TN has been highlighted previously, which demonstrate elevation of multiple inflammatory biomarkers in different samples of TN patients, such as serum and cerebrospinal fluid (CSF) (14).

This literature review aims to summarize current clinical and laboratory findings along the perioperative timeline to find out biomarkers for clinical use, and discuss underlying molecular mechanisms for a better understanding of the role of inflammation in TN and directions for future clinical trials and basic research.

## 2 Methods

A search of published articles was performed through PubMed database using strategy (trigeminal neuralgia[Title/Abstract]) AND (inflammatory[Title/Abstract]), and a search of registered clinical trials was performed through Cochrane Central Register of Controlled Trials (CENTRAL) database using ‘trigeminal neuralgia’ as key word, with a date range of 2000 to 2025. A total of 142 English articles was acquired from PubMed, and a total of 95 clinical trials registered on clinicaltrials.gov was acquired for further screening. Original articles of both human and animal studies related to ‘trigeminal neuralgia’, ‘surgical intervention’ and ‘inflammatory biomarkers’, as well as registered trials related to ‘trigeminal neuralgia’ and ‘surgical intervention’ were included, whereas articles in the format of reviews, systematic reviews and/or meta-analyses, case reports or case series, commentaries, editorials, letters, as well as articles irrelevant to or not fully covering (e.g., comparing the surgical interventions without analyzing inflammatory biomarkers, or studying inflammatory biomarkers in non-surgical scenarios) this topic were excluded.

## 3 Human participants

For recurrent cases and patients refractory to pharmacotherapies, surgical treatments may be contemplated. Typical surgical treatments for TN include peripheral neurectomy, microvascular decompression (MVD), percutaneous balloon compression (PBC) and stereotactic radiosurgery (SRS).

### 3.1 Peripheral neurectomy

Peripheral neurectomy is a simple, low-risk and well-tolerated procedure of transection that can be performed on all terminal branches of the trigeminal nerve. Previous study was established on 12 males and 8 females with an age range of 35–68 years demonstrated that peripheral neurectomy exerted a positive effect on alleviating pain in TN patients, with an average pain relief period of 24–33 months after surgery (15). This method may be particularly suitable for elderly or debilitated patients in whom other invasive neurosurgical interventions are contraindicated. After studying the biopsy specimens collected from 4 male and 7 female elderly TN patients with an average age of 63.64 ± 6.15 and without underlying cause, it demonstrated that this procedure solved the consequences including axonal interconnection, spontaneous activity and ectopic impulse conduction, suggesting the vascular pathologic alterations of peripheral neurovascular bundle as the primary factor in pathogenesis of TN in this cohort (16).

### 3.2 MVD

The MVD surgery at the REZ of the trigeminal nerve is the most definitive surgical treatment for TN, since arterial compression of the trigeminal nerve is the most often cause of TN. It is non-destructive because the nerve is merely decompressed of conflicting blood vessels during open fossa posterior surgery. Therefore, MVD has been considered as the first-line surgery in medically refractory patients especially for those with classical TN (8).

### 3.3 PBC

Percutaneous compression by a balloon is a destructive mechanical procedure, which is performed at the level of TG of patients with TN. PBC is overall a safe and effective method, especially being an option for those who is in poor general condition and refuses to receive craniotomy. Pooled analyses reported that 55–80% of patients (*n* = 755) with TN were pain-free after PBC, with a follow-up of 4–11 years (17).

### 3.4 SRS

Radiosurgery has been utilized since the 1950s to treat refractory TN, with Lars Leksell being the first to target the trigeminal nerve using focused radiation. SRS uses 3D imaging to target high doses of radiation to the affected area by damaging the DNA of the targeted cells, but with minimal impact on the surrounding tissues. As one type of SRS, gamma knife surgery (GKS) is the only non-invasive but destructive surgical alternatives for the treatment of drug-resistant TN, by targeting the trigeminal REZ to disrupt the pain signals. Its safety and efficacy has been verified in a large population of 225 male and 272 female TN patients (total *n* = 497) with a median age of 68.3 years and with a clinical follow-up of at least 1 year (18).

## 4 Animal models

### 4.1 Chronic constriction injury of the infraorbital nerve model

The CCI-ION model is the most widely used animal model to study TN (19), as it results in the development of facial sensory alterations, including heat and mechanical hyperalgesia, by mimicking the trigeminal nerve compression and demyelination in clinical TN patients (20). In particular, this model can induce significant and stable spontaneous pain in rodents, including increased facial grooming behavior, increased pain threshold of mechanical stimulation and heat radiation stimulation in the ipsilateral area (21). In this type of model, the TN-related pain and the analgesic profile of certain kind the therapies could be assessed in different time periods (22) and evaluated by the change in ipsilateral mechanical threshold (23).

### 4.2 Distal ligation of the infraorbital nerve model

The traditional CCI-ION model has several side effects such as difficulty in exposing the surgical field and ocular damage in rats, leading to increased grooming activities. Therefore, an alternative model, the distal ligation of the infraorbital nerve (dION) was established to simulate the symptoms of TN in rats (24). The dION model avoided the pressure that may induce eye discomfort and subsequent resultant behavioral presentation, thus making it a more relevant nerve injury instead of a mixed nerve/tissue injury as compared to previous models do (24).

### 4.3 Partial infraorbital nerve transection model

The TN model could also be created by the intraoral partial transection of the infraorbital nerve surgery, which induces long-lasting orofacial thermal hyperalgesia (25). In this model, microglial activation after p-IONX was transmitter from medullar to lumber dorsal horn in a time-dependent manner, and the pain sensitization derived from microglial activation may be attenuated at early but not late stage after p-IONX (25).

### 4.4 Cobra venom-treated model

Another experimental animal model for TN has been developed by injecting cobra venom into the infraorbital nerve trunk, which generates mechanical allodynia 3 days after surgery and lasts for 60 days after injection at the ipsilateral side (26). Meanwhile, the neurogenic inflammatory responses were observed in the affected receptive field (27). This is a suitable model to investigate the TN-induced cognitive deficits by performing behavioral tests such as Morris Water Maze (28).

### 4.5 Complete Freund adjuvant-injected model

The CFA-induced TN model is developed by injecting with CFA on the unilateral face of the rodents to stimulate percutaneous trigeminal nerve (29). This model exhibits greater mechanical but similar heat hypersensitivity compared to its counterparts, and cognitive impairments in novel object exploration, as well as in social and spatial memory have also been detected (29).

### 4.6 Animal models for SRS

The effects of SRS to the trigeminal nerve at a cellular level were previously described in a primate model. In this model, a dose of 80Gy caused focal axonal degeneration without inflammatory changes, whereas a nerve necrosis was observed after a dose of 100Gy (30). When applying two different radiosurgical doses (40Gy or 80Gy) to the lumbar dorsal root ganglion (DRG) in rats, the expression of glial fibrillary acidic protein (GFAP), a marker for activated satellite glial cells, was inhibited (31).

## 5 Preoperative changes in inflammatory factors

Since inflammation plays a critical role in the pathogenesis and progression of TN, understanding how inflammatory factors change in biological specimens from clinical patients before and after surgical interventions may help us to determine the severity of disease, efficacy of surgical treatment and long-term follow-up for safety and prognosis. In addition, targeting these cytokines and chemokines may be capable of suppressing activation of glial cells and alleviating chronic neuropathic pain including TN (32). Currently, only limited number of clinical studies have been established, and therefore, results from animal studies could assist us in digging out the molecular mechanisms to explain these alterations. The promising findings in large-scaled clinical trials and repeatably verified conclusions may allow the development of trustful biomarkers for clinical use. The perioperative alterations in these inflammatory biomarkers are summarized in Table 1.

**Table 1:** Changes in levels of inflammatory biomarkers in TN patients undergoing surgical interventions.

Groups	Sample source	Biomarker	Preoperative change	Postoperative change	Conclusion	Ref.
Microvascular decompression (MVD)						
① Primary TN patients submitted to MVD (*n* = 44) ② HFS patients (*n* = 47) ③ Healthy individuals (*n* = 28)	Serum	IL-1β	↑↑↑ (① vs. ③)	/	Vascular compression in TN induces an increase in a variety of cytokines.	(34) (2019)
		IL-6	↑↑↑ (① vs. ③)	/		
		IL-8 (CXCL8)	↑↑↑ (① vs. ③)	/		
		TNF-ɑ	↑↑↑ (① vs. ③)	/		
① TN patients submitted to MVD (*n* = 27) ② Healthy individuals (*n* = 11) ③ Individuals without neurological diseases (*n* = 23) ④ Individuals undergoing routine surgical procedures (*n* = 28)	CSF	TNF-β	↑↑↑	⇔	TNF-β did not seem to change after surgery.	(14) (2019)
		TRAIL	↑↑↑	↓↓↓	TRAIL level in TN may be reversible by surgery.	
① TN patients submitted to MVD (*n* = 8) ② Patients with minor urological disorders undergoing routine surgical procedures (*n* = 2)	CSF collected one day before MVD	Apolipoproteins	↑↑↑	/	Inflammation components participate in the in the pathophysiology of TN.	(60) (2020)
		CO8B, CO8G, C5 (proteins involved in the complement system)	↑↑↑	/		
① TN patients submitted to MVD (*n* = 30) ② TN patients submitted to PBC (*n* = 30)	Serum	IL-1β	⇔	↓↓↓	MVD is effective to significantly reduce the pro-inflammatory cytokines at postoperative 3 days and 5 days compared with preoperation.	(65) (2020)
		IL-6	⇔	↓↓↓		
		TNF-ɑ	⇔	↓↓↓		
① TN patients (*n* = 10) ② Healthy individuals (*n* = 10)	Saliva	IL-1β	↑↑↑	/	Increased levels of chemokines play a vital role in TN pathogenesis.	(35) (2021)
		TNF-ɑ	↑↑↑	/		
		CCL2	↑↑↑	/		
		IL-17A	↑↑↑	/		
		IL-6	↑↑↑	/		
		IL-8 (CXCL8)	↑↑↑	/		
① TN patients submitted to MVD (*n* = 20) ② TN patients submitted to PBC (*n* = 20)	Serum	IL-6	⇔	↑↑↑ (compared to preoperative value in MVD group)	Greater surgical trauma from MVD.	(66) (2022)
① TN patients submitted to MVD (*n* = 8) ② Control participants undergoing MVD for HFS (*n* = 2) ③ Control participants with NPH (*n* = 2)	CSF collected during MVD	IL-18, IL-33 and IL-9 (pro-inflammatory cytokines)	↑↑↑	/	Neuro-inflammation is present in TN.	(49) (2023)
		RANTES and ENA-78 (chemokines)	↑↑↑	/		
		TRAIL and sCD40L (TNF superfamily)	↑↑↑	/		
		EGF, PDGF-AB/BB, FGF-2 (growth factors)	↑↑↑	/		
Percutaneous balloon compression (PBC)						
① TN patients submitted to MVD (*n* = 30) ② TN patients submitted to PBC (*n* = 30)	Serum	IL-1β	⇔	↓↓↓	PBC is effective to significantly reduce the pro-inflammatory cytokines at postoperative 3 days and 5 days compared with preoperation.	(65) (2020)
		IL-6	⇔	↓↓↓		
		TNF-ɑ	⇔	↓↓↓		
① TN patients submitted to MVD (*n* = 20) ② TN patients submitted to PBC (*n* = 20)	Serum	IL-6	⇔	↑	Postoperative levels of IL-6 was significantly increased in MVD group but not in PBC group.	(66) (2022)

C5, Complement C5; CO8B, Complement component C8 beta chain; CO8G, Complement component C8 gamma chain; CSF, cerebrospinal fluid; HFS, hemifacial spasm; MVD, microvascular decompression; NPH, normal pressure hydrocephalus; TN, trigeminal neuralgia; TNF-β, tumor necrosis factor beta; TRAIL, TNF-related apoptosis inducing ligand. ↑↑↑, significantly increased; ↑, increased without significance; ↓↓↓, significantly decreased; ⇔, unchanged or within normality range; /, not mentioned.

### 5.1 Interleukin (IL)-1β

The pro-inflammatory cytokine IL-1β acts as an important mediator of the inflammatory response, and its induction of cyclooxygenase-2 (COX2) in the central nervous system is believed to contribute to inflammatory pain hypersensitivity (33). It was shown in an observational cohort study that preoperative serum levels of IL-1β was significantly increased in TN patients (*n* = 44) compared to healthy volunteers (*n* = 28) (34). IL-1β was also found elevated in the saliva of TN subjects (*n* = 10) compared to normal controls (*n* = 10) (35).

The levels of IL-1β have also been studied in animal models of TN. For instance, in CCI-ION (36, 37), dION (38) and CFA (39–41) models, IL-1β has been identified consistently overexpressed at both protein and mRNA levels in TG.

### 5.2 Il-6

Another cytokine, IL-6, is not only promptly and transiently produced in response to infections and injuries for host defense, but also plays a pathological effect on chronic inflammation and autoimmunity if dysregulated continual synthesis exists (42). IL-6 was observed to be significantly elevated in both serum (34) and saliva (35) specimens of TN patients, and statistically related to the pain sensations associated with neuropathic pain (43), indicating that IL-6 may play a critical role in the signaling pathways generating ectopic impulses from these cranial nerves.

In TN models, the expression of IL-6 was found significantly increased in the TG of both the CCI-ION (36) and the p-IONX (44) models. Furthermore, the overexpression of IL-6 mRNA in TG of the CCI-ION rats was detected as early as 1.5 h after surgery, with mRNA levels even higher than at later postoperative times, which showed a different response of sensory ganglia cells to injury in sciatic nerve (SN)-CCI rats (45).

### 5.3 Il-8

IL-8 (also called CXCL8) is the most potent chemokine recruiting neutrophils to the site of damage or infection, which is called chemotaxis (46). In the aforementioned studies, IL-8 was shown to be another up-regulated inflammatory factor in the serum of patients with primary TN scheduled for MVD (34) and in the saliva of TN patients (35) compared to healthy controls.

### 5.4 Il-17A

IL-17A is a proinflammatory cytokine produced by activated T cells and stimulates the expression of IL-6 and COX2 (47). Compared to normal controls, saliva levels of IL-17A have been detected increased in individuals with TN (35).

### 5.5 Il-18

IL-18, which belongs to the IL-1 superfamily and a powerful inducer of IFN-ɣ, is a potent pro-inflammatory cytokine participating in host defense against infections and in the regulation of the innate and acquired immune response (48). In a clinical study with very small sample size, IL-18 was found to be significantly increased together with another two pro-inflammatory cytokines (IL-33 and IL-9) in the CSF specimen collected during the MVD procedure of patients with TN (*n* = 8) compared to individuals undergoing MVD for hemifacial spasm (HFS; *n* = 2) or participants with normal pressure hydrocephalus (NPH; *n* = 2) (49), suggesting the presence of neuroinflammation in TN.

In the CFA mouse model of TN pain have been linked to a significant increase in IL-18 mRNA expression in TG neurons (40).

### 5.6 Tumor necrosis factor alpha

TNF sits in a central position in inflammatory reactions, driving inflammatory responses not only by inducing inflammatory gene expression, but also by inducing cell death (50). TNF-ɑ was found to be increased in the serum (34) and saliva (35) specimens of TN patients.

In addition, animal studies also revealed that overexpressed TNF-ɑ was detected in the TG of CCI-ION models (35, 36), as well as in CFA-induced model (51).

### 5.7 Tumor necrosis factor beta

After analyzed the levels of 92 protein biomarkers related to inflammation using the proximity extension assay technology, the protein level of TNF-β (also known as lymphotoxin-ɑ) in the lumbar CSF was significantly higher in patients with TN (*n* = 27) compared to all 3 control groups [healthy control (*n* = 11), individuals without neurological diseases (*n* = 23) and individuals undergoing routine surgical procedures (*n* = 28)] (14).

### 5.8 TNF-related apoptosis inducing ligand

TRAIL, which belongs to the tumor necrosis factor superfamily, is a cytokine functioning as a ligand that binds to certain death receptors and induces apoptosis primarily in tumor cells. Preoperatively, the CSF level of TRAIL was also found to be up-regulated in TN patients compared to control groups (14).

### 5.9 RANTES/CCL5

Proinflammatory chemokine RANTES (also known as CCL5) is upregulated and attracts T cells in inflammatory conditions, leading to their proliferation and activation and further the prolongation of the inflammatory responses (52). The CSF level of RANTES should be very low in healthy individuals, however, it was found increased dramatically at the onset and progression of MS (53, 54). RANTES was highly overexpressed in fatty degenerated jawbone (FDOJ) tissues of 15 patients with atypical facial pain (AFP) and TN, which was diminished by surgical debridement of FDOJ areas and followed by a reduced chronic facial pain, suggesting that the diminishment of RANTES overexpression by surgical interventions may resolve chronic neuropathic pain (55).

### 5.10 CCL2

The C-C motif chemokine ligand 2 (CCL2) is essential for glial cell activation inflammatory and neuropathic pain, which could be utilized as a therapeutic target for neuropathic pain (56, 57). Elevated levels of chemokine CCL2 have been observed in the saliva from TN subjects compared with controls (35).

In animal models, the levels of CCL2 were increased significantly in the TNC region of the CCI-ION rats (58). In fact, CCL2 seemed to be involved in the early events accompanying the ION lesion rather than in long-term alterations and the maintenance of trigeminal mechanical hypersensitivity in the CCI-ION model (59).

### 5.11 Apolipoproteins

When comparing with control participants undergoing minor urological surgery, a significant increase in apolipoproteins (including APOC2, APOA4, APOM and APOA1) and proteins involved in the complement cascade (including CO8B, CO8G and C5) has been detected preoperatively in the CSF of TN patients scheduled for MVD, suggesting an inflammatory component in the pathophysiology of TN (60).

## 6 Postoperative changes in inflammatory factors

Notably, inflammatory response triggered by foreign materials is often seen in patients receiving surgical treatments. Traditional decompressive methods for TN patients involve placing foreign material (most often Teflon) between the affected nerve and the compressive blood vessel to maintain distance between the two structures. However, inflammatory reactions related to the foreign material have been reported in numerous papers, resulting in granuloma formation and recurrence of pain (61–63). Therefore, the tentorial slinging technique without using foreign material has been developed as an alternative decompressive method to minimize the inflammatory response and lower the rate of trigeminal pain recurrence over time (64). Postoperative changes of inflammatory factors have been explored by several studies, with results sometimes being contradicting, partially due to heterogeneities among these limited number of studies.

### 6.1 Il-1β

In a retrospective review of elderly primary TN patients undergoing MVD (*n* = 30) or PBC (*n* = 30), the results showed that the serum levels of IL-1β were not significantly different between the two surgery groups preoperatively, while IL-1β dropped dramatically in both groups at postoperative day 3 and day 5, indicating that IL-1β may act as an indicator for the effectiveness of PBC (65).

### 6.2 Il-6

When comparing the preoperative and the postoperative serum levels of a variety of biomarkers in one cohort study, IL-6, an acute phase inflammatory marker, was dramatically increased in patients with drug-resistant TN at 2 days after MVD (*p* = 0.013), and holding a trend toward increase in those after PBC (*p* = 0.069) (66), indicating that a greater surgical trauma related to the craniotomy procedure of MVD may sustain a stressful condition for the organism that responds by implementing the mechanisms of inflammation (67). However, contradictory observations have been reported in another retrospective study, showing a significant postoperative reduction in IL-6 at 3 days and 5 days after MVD and PBC surgical treatments (65).

### 6.3 TNF-ɑ

In the above-mentioned retrospective study comparing TN patients undergoing MVD and PBC, the serum levels of TNF-ɑ were not significantly different between the two surgery groups preoperatively, but dropped dramatically in both groups at postoperative day 3 and day 5 (65).

### 6.4 TNF-*β*

The CSF level of TNF-β remained unchanged after MVD, suggesting that it may act as a constitutive marker for TN (14). This could be explained by the fact that TNF-β participates in the pathophysiology of the disease via disrupting oligodendrocytes and causing demyelination.

### 6.5 Trail

The elevated CSF level of TRAIL before MVD dropped significantly at 10–30 months after surgery to a level toward control groups, and such an MVD-related normalization suggested that TRAIL may be used as an indicator for the successfulness of this surgical intervention for TN (14). However, when comparing to pain-free controls with HFS also undergoing MVD, the levels of TRAIL together with sCD40L, another member in the TNF superfamily, were significantly elevated in the CSF collected during MVD (49).

### 6.6 RANTES/CCL5

As compared with 4 pain-free controls, the chemokines (RANTES and ENA-78) were significantly higher in CSF of 8 medically refractory TN patients collected during MVD (49).

## 7 Essential mechanisms and future research

**Figure 1:**
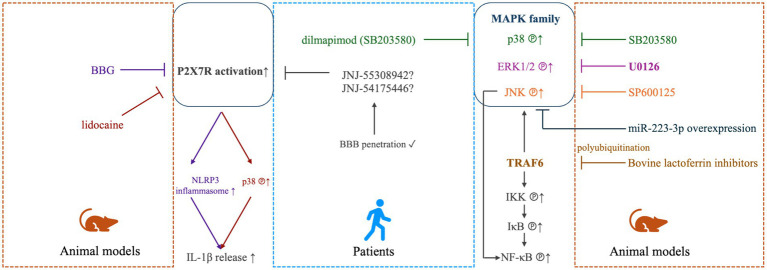
MAPK- and P2X7-mediated modulation in TN. In TN animal models (right panel), elevated phosphorylation of mitogen-activated protein kinase (MAPK) family, including extracellular signal-regulated kinase 1/2 (ERK1/2), p38 and c-Jun N-terminal kinases (JNK) was detected to be linked with inflammatory and neuropathic pain, which could be effectively alleviated by antagonists of ERK1/2 (U0126), p38 (SB203580) or JNK (SP600125) (70), or miR-223-3p overexpression (72). The MAPK signaling may partially be mediated by the polyubiquitinated TNF receptor-associated factor 6 (TRAF6), which also facilitates the activation of transcriptional factor nuclear factor-κB (NF-κB) via inducing phosphorylation of IκB kinase (IKK) and IκB. TRAF6 may also aggravate neuropathic pain by activating the c-Jun/NF-κB signaling pathway (74). The polyubiquitination of TRAF6 was attenuated by bovine lactoferrin inhibitors in a trigeminal nerve injury rat model (71). For TN patients (middle panel), dilmapimod (SB681323), a selective p38MAPK inhibitor, was verified to be effect in neuropathic pain control (75). In animal models (left panel), The P2X7/NLRP3/IL-1β and P2X7/p38/IL-1β pathways have also been shown to contribute to the occurrence and development of neuropathic pain, which could be targeted by brilliant blue G (BBG) (38) and lidocaine (77), respectively. In humans (middle panel), newly-developed P2X7R inhibitors, such as JNJ-55308942 and JNJ-54175446, may be more promising antagonists due to their ability of BBB penetration (78, 79).

The pro-inflammatory cytokines, such as IL-1β, IL-6 and TNF-ɑ, are essential for the persistent activation of microglia and astrocytes in the spinal trigeminal nucleus, which is important in the establishment and maintenance of TN (57). The release of these inflammatory factors observed in patients with TN may be caused by focal nerve demyelination and vascular compression of the trigeminal nerve roots, as verified by evidence obtained from TN animal models (68, 69). At molecular level, the significant increases of these pro-inflammatory cytokines and chemokines have been associated with the up-regulated phosphorylation of the mitogen-activated protein kinases (MAPKs), which includes extracellular signal-regulated kinase (ERK), p38 and c-Jun N-terminal kinase (JNK; Figure 1). These singling molecules may be activated in the TG neurons to participate in the regulation of TN-related pain (39, 70, 71), and antagonizing them could be a practical strategy to reverse the pain (58, 72). For instance, in the injured trigeminal nerve of the CCI-ION model, it was shown that stem cells from human exfoliated deciduous teeth transplantation at the lesion site led to reduced inflammatory cell infiltration and pro-inflammatory cytokine release (TNF-ɑ and IL-1β). Moreover, the administration of resveratrol reversed the activation of astrocytes and microglia by downregulating the phosphorylated MAPKs in an adenosine monophosphate activated protein kinase (AMPK)-dependent manner (73). The MAPK signaling may partially be mediated by the polyubiquitinated TNF receptor-associated factor 6 (TRAF6), which also facilitates the activation of transcriptional factor nuclear factor-κB (NF-κB). Meanwhile, TRAF6 may also aggravate neuropathic pain by activating the c-Jun/NF-κB signaling pathway (74). The polyubiquitination of TRAF6 was attenuated by bovine lactoferrin inhibitors in a trigeminal nerve injury rat model (71). However, the clinical evidence for treatment efficacy of these MAPK-related antagonists in TN patients was very limited, only dilmapimod (a p38MAPK inhibitor) has been reported as a promising compound in neuropathic pain control (75). In addition to the MAPK-associated signaling pathways, the increased release of the pro-inflammatory factors (TNF-ɑ and IL-1β) could also be modulated by the activation of the colony-stimulating factor 1 (CSF1) - colony-stimulating factor 1 receptor (CSF1R) pathway, which regulates pain development in TG (44). Furthermore, the IL-1β maturation was promoted, the IL-1β released by microglia was increased, and the neuroinflammatory response and neuropathic pain in TN models was developed through the activation of P2X7/NLRP3/IL-1β or the P2X7/p38/IL-1β pathways (38, 76, 77), which could be blocked by brilliant blue G (BBG) (38) and lidocaine (77), respectively, both are inhibitors of the P2X7 receptor. Several P2X7R antagonists, e.g., AstraZeneca’s AZD9056 and Pfizer’s CE-224535 for rheumatoid arthritis, GlaxoSmithKline’s GSK-1482160 for chronic inflammatory pain have been tested in clinical trials but none have reached final approval due to lack of efficacy. Newly developed compounds, such as JNJ-55308942 and JNJ-54175446, may be more promising antagonists due to their ability of BBB penetration (78, 79). These signaling pathways might be novel targets for the treatment of TN, in either pharmacological or non-pharmacological way. Various mechanisms have been suggested based on animal findings, yet gaps still remain on translation from animal studies to human ones, partially due to less heterogeneities in these animal models. The greater heterogeneity in human requires validation in multi-centered larger cohorts. In addition, the insufficiency in replicating the full breadth of etiological factors reported in corresponding clinical studies of TN, including the absence of discovered TN-related human channel mutations in animal models, as well as reduced levels of substance P and calcitonin gene-related peptide (CGRP) in medullary dorsal horn of animal models following trigeminal nerve compression, have been detailedly discussed in a previous review (80).

By searching randomized controlled trials (RCTs) registered on clinicaltrials.gov through the CENTRAL database, we noticed that the number of registered trials testing surgical treatments for TN was low (Table 2). Most of these RCTs were designed for pharmacological treatments or radiofrequency therapies. Surprisingly, no registered trial involved inflammatory biomarkers as one of the outcome measurements. They predominantly focused on the improvement in main symptoms, such as pain and numbness. Due to the important role inflammation plays in the progression of TN, and based on the current findings of the alterations of inflammatory biomarkers in TN patients who are submitted to surgical treatments, we suggest to include the measurement of inflammatory factors in the design of future clinical studies. Given the dynamic change of these potential biomarkers with time, determination of the best observation window for these biomarkers was needed in future studies. Additionally, clinical evidence is also required for surgical interventions other than MVD and PBC, such as peripheral neurectomy and SRS, both before and after the surgical treatments, to build up the solid knowledge whether and which biomarkers are suitable ones to use in clinical practice. Longer follow-up period and further clinical investigation on other inflammatory biomarkers are also required in future research.

**Table 2:** Registered RCTs testing the application of surgical interventions in patients with TN.

^*^ID	Title	Sponsor	Participants	Groups	Outcome	Status
NCT 03991039	Comparing Clinical Benefits of Gamma Knife and Microvascular Decompression for Trigeminal Neuralgia	University of Baghdad	Primary idiopathic TN (children and adults)	① Gamma knife (80Gy) ② MVD	BNI-PS VAS BPI	Completed
NCT 02427074	Comparison Between Radiofrequency and Balloon Compression in the Treatment of Idiopathic Trigeminal Neuralgia	University of São Paulo	Idiopathic TN (adults)	① Balloon Compression Rhizotomy ② Radiofrequency Thermal Coagulation Rhizotomy	NRS	Terminated
NCT 05810428	Artificial Intelligence to Predict Surgical Outcomes and Assess Pain Neuromodulation in Trigeminal Neuralgia Subjects	IRCCS San Raffaele	TN (adults)	① Gamma knife Radiosurgery + virtual reality rehabilitation ② Gamma knife Radiosurgery ③ Healthy controls	NRS BNI-PS	Recruiting
NCT 04117035	A Personalized Radiosurgery Procedure for People with Trigeminal Neuralgia to Improve Pain, Quality of Life and Reduce Complications	University of Leeds	Idiopathic TN or MS-related TN (adults)	① Personalized Gamma knife Radiosurgery ② Gamma knife Radiosurgery with standard care	BNI-PS BNI-NS	Recruiting
NCT 03152955	Postoperative Analgesia in Patients with Microvascular Decompression	Xiangya Hospital of Central South University	TN patients scheduled for MVD (adult)	① Scalp nerve block and patient-controlled analgesia ② Patient-controlled analgesia (sufentanil, ondansetron and ketamine) ③ Patient-controlled analgesia (sufentanil, ondansetron)	VAS	Unknown

*This refers to the ClinicalTrials.gov ID. BNI-PS, Barrow Neurological Institute Pain Intensity Scale; BNI-NS, Barrow Neurological Institute Numbness Intensity Scale; BPI, Brief Pain Inventory; MVD, microvascular decompression; NRS, numeric rating scale; VAS, Visual Analog Scale.

## 8 Conclusion and future perspectives

**Figure 2:**
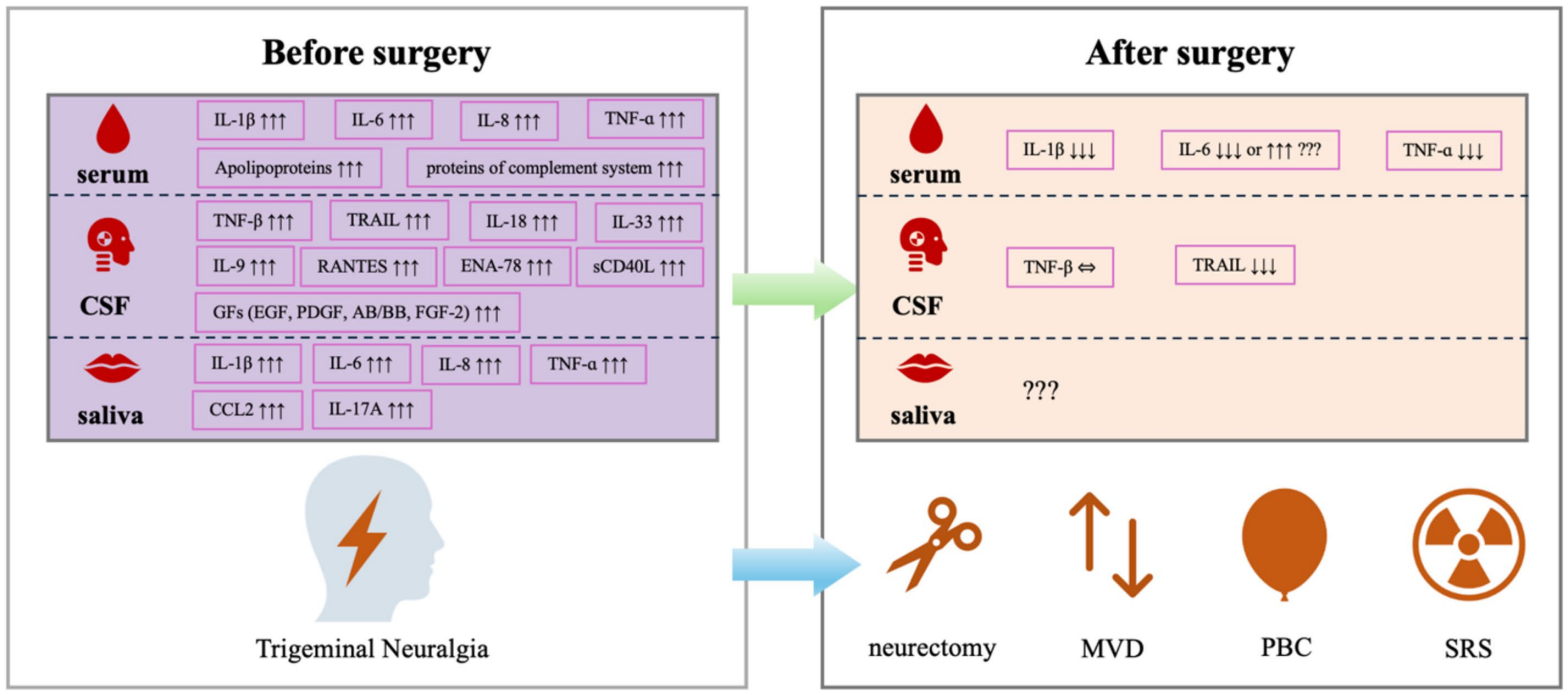
Alterations in inflammatory biomarkers in patients with TN before and after the surgical treatments.

By reviewing the previously reported work on changes in inflammatory factors in TN patients submitted to typical surgical treatments include peripheral neurectomy, MVD, PBC and SRS, as well as the results from TN animal models, this narrative review revealed that pro-inflammatory cytokines and chemokines, such as IL-1*β*, IL-6, TNF-ɑ reached high levels in serum, CSF or saliva specimens from TN patients, which could be reversed by PBC, but not always by MVD. The elevated preoperative level of TRAIL was reversable by MVD, but the elevated preoperative level of TNF-β was not (Figure 2). Such different patterns responding to different surgical interventions are of research interest and require further investigations.

Currently, only a limited number of registered RCTs testing the application of surgical interventions in TN patients are being performed, and none of them have incorporated inflammatory biomarkers as endpoints (Table 2). The gap between inflammatory biomarkers and surgical outcomes need to be filled in future trial design. The results discussed in this review provided valuable information for further exploration and would need to be verified in larger-scaled clinical studies. Of note, the timing and intervals for sampling varied across studies, leading to heterogeneities among studies. Observation of dynamic changes of these markers after surgery in the future would contribute to standardization of timing and interval of postoperative samplings. Pain relief may compose major observation outcome, but other parameters may also be explored. In addition, the analytical platforms and statistical approaches also need to be more standardized for biomarkers that have been tested and shown changes in human studies such as IL-1*β*, IL-6, TNF-ɑ, TRIAL and TNF-β, as well as MAPK or P2X7 pathway-associated proteins in future studies. Furthermore, comparisons of peripheral blood, CSF and saliva are also needed to optimize sample selections. The alterations in these inflammatory biomarkers were modulated by a variety of signaling pathways, including MAPK- or P2X7-associated pathways, a better understanding of these underlying molecular mechanisms may help in the development of novel treatments for patients with TN.

## Glossary

AMPK: adenosine monophosphate activated protein kinase
CCI-ION: chronic constriction injury of the infraorbital nerve
CCL2: C-C motif chemokine ligand 2;
CFA: complete Freund adjuvant
CSF1: colony-stimulating factor 1
COX2: cyclooxygenase-2
CSF: cerebrospinal fluid
dION: distal ligation of the infraorbital nerve
DRG: dorsal root ganglion
ERK: extracellular signal-regulated kinase
GKS: gamma knife surgery
IL: interleukin
JNK: c-Jun N-terminal kinase
MAPKs: mitogen-activated protein kinases
MS, multiple sclerosis
MVD: microvascular decompression
NVC: neurovascular compression
PBC: percutaneous balloon compression
p-IONX: partial infraorbital nerve transection
RCT: randomized controlled trial
REZ: root entry zone
SRS: stereotactic radiosurgery
TG: trigeminal ganglion
TN: trigeminal neuralgia
TNF: tumor necrosis factor
TRAIL: TNF-related apoptosis inducing ligand

## References

[1] Headache classification Committee of the International Headache Society (IHS) the international classification of headache disorders, 3rd edition. Cephalalgia. (2018) 38:1–211. doi: 10.1177/0333102417738202, PMID: 29368949

[2] Lynds R Lyu C Lyu GW Shi XQ Rosen A Mustafa K. Neuronal plasticity of trigeminal ganglia in mice following nerve injury. J Pain Res. (2017) 10:349–57. doi: 10.2147/JPR.S120092, PMID: 28223844 PMC5310634

[3] Chen Q Yi DI Perez JNJ Liu M Chang SD Barad MJ. The molecular basis and pathophysiology of trigeminal neuralgia. Int J Mol Sci. (2022) 23:23. doi: 10.3390/ijms23073604, PMID: 35408959 PMC8998776

[4] Cruccu G Di Stefano G Truini A. Trigeminal neuralgia. N Engl J Med. (2020) 383:754–62. doi: 10.1056/NEJMra1914484, PMID: 32813951

[5] Peker S Kurtkaya O Uzun I Pamir MN. Microanatomy of the central myelin-peripheral myelin transition zone of the trigeminal nerve. Neurosurgery. (2006) 59:354–9. doi: 10.1227/01.NEU.0000223501.27220.69, PMID: 16883175

[6] MacDonald BK Cockerell OC Sander JW Shorvon SD. The incidence and lifetime prevalence of neurological disorders in a prospective community-based study in the UK. Brain. (2000) 123:665–76. doi: 10.1093/brain/123.4.665 10733998

[7] Mueller D Obermann M Yoon MS Poitz F Hansen N Slomke MA. Prevalence of trigeminal neuralgia and persistent idiopathic facial pain: a population-based study. Cephalalgia. (2011) 31:1542–8. doi: 10.1177/0333102411424619, PMID: 21960648

[8] Bendtsen L. Advances in diagnosis, classification, pathophysiology, and management of trigeminal neuralgia. Lancet Neurol. (2020) 19:784–96. doi: 10.1016/S1474-4422(20)30233-7, PMID: 32822636

[9] De Toledo IP. Prevalence of trigeminal neuralgia: a systematic review. J Am Dent Assoc. (2016) 147:e2:570–6. doi: 10.1016/j.adaj.2016.02.014 27017183

[10] Maltez N. Trigeminal neuralgia in systemic sclerosis. Semin Arthritis Rheum. (2021) 51:318–23. doi: 10.1016/j.semarthrit.2021.01.001, PMID: 33461050

[11] Lutz J Thon N Stahl R Lummel N Tonn JC Linn J. Microstructural alterations in trigeminal neuralgia determined by diffusion tensor imaging are independent of symptom duration, severity, and type of neurovascular conflict. J Neurosurg. (2016) 124:823–30. doi: 10.3171/2015.2.JNS142587, PMID: 26406792

[12] DeSouza DD Hodaie M Davis KD. Abnormal trigeminal nerve microstructure and brain white matter in idiopathic trigeminal neuralgia. Pain. (2014) 155:37–44. doi: 10.1016/j.pain.2013.08.029, PMID: 23999058

[13] Smith CA Paskhover B Mammis A. Molecular mechanisms of trigeminal neuralgia: a systematic review. Clin Neurol Neurosurg. (2021) 200:106397. doi: 10.1016/j.clineuro.2020.106397, PMID: 33338828

[14] Ericson H. Cerebrospinal fluid biomarkers of inflammation in trigeminal neuralgia patients operated with microvascular decompression. Pain. (2019) 160:2603–11. doi: 10.1097/j.pain.0000000000001649, PMID: 31373951

[15] Chandan S Halli R Sane VD. Peripheral neurectomy: minimally invasive surgical modality for trigeminal neuralgia in Indian population: a retrospective analysis of 20 cases. J Maxillofac Oral Surg. (2014) 13:295–9. doi: 10.1007/s12663-013-0512-9, PMID: 25018603 PMC4082550

[16] Sahoo NK Thakral A Deb P Roy ID. Histopathological evaluation of inferior alveolar neurovascular bundle in cases of trigeminal neuralgia. J Maxillofac Oral Surg. (2020) 19:54–60. doi: 10.1007/s12663-019-01214-z, PMID: 31988565 PMC6954937

[17] Bendtsen L Zakrzewska JM Abbott J Braschinsky M Di Stefano G Donnet A. European academy of neurology guideline on trigeminal neuralgia. Eur J Neurol. (2019) 26:831–49. doi: 10.1111/ene.13950, PMID: 30860637

[18] Regis J Tuleasca C Resseguier N Carron R Donnet A Gaudart J. Long-term safety and efficacy of gamma knife surgery in classical trigeminal neuralgia: a 497-patient historical cohort study. J Neurosurg. (2016) 124:1079–87. doi: 10.3171/2015.2.JNS142144, PMID: 26339857

[19] Deseure K Hans GH. Chronic constriction injury of the rat's infraorbital nerve (IoN-CCI) to study trigeminal neuropathic pain. J Vis Exp. (2015) 103:e53167. doi: 10.3791/53167, PMID: 26437303 PMC4692620

[20] Chichorro JG Zampronio AR Cabrini DA Franco CR Rae GA. Mechanisms operated by endothelin ETA and ETB receptors in the trigeminal ganglion contribute to orofacial thermal hyperalgesia induced by infraorbital nerve constriction in rats. Neuropeptides. (2009) 43:133–42. doi: 10.1016/j.npep.2008.12.001, PMID: 19157542

[21] Vos BP Hans G Adriaensen H. Behavioral assessment of facial pain in rats: face grooming patterns after painful and non-painful sensory disturbances in the territory of the rat's infraorbital nerve. Pain. (1998) 76:173–8. 9696471 10.1016/s0304-3959(98)00039-6

[22] von Hehn CA Baron R Woolf CJ. Deconstructing the neuropathic pain phenotype to reveal neural mechanisms. Neuron. (2012) 73:638–52. doi: 10.1016/j.neuron.2012.02.008, PMID: 22365541 PMC3319438

[23] Yang YJ Hu L Xia YP Jiang CY Miao C Yang CQ. Resveratrol suppresses glial activation and alleviates trigeminal neuralgia via activation of AMPK. J Neuroinflammation. (2016) 13:84. doi: 10.1186/s12974-016-0550-6, PMID: 27093858 PMC4837542

[24] Ding W. An improved rodent model of trigeminal neuropathic pain by unilateral chronic constriction injury of distal infraorbital nerve. J Pain. (2017) 18:899–907. doi: 10.1016/j.jpain.2017.02.427, PMID: 28238950 PMC5537008

[25] Zhang SH. Widespread pain sensitization after partial infraorbital nerve transection in MRL/MPJ mice. Pain. (2016) 157:740–9. doi: 10.1097/j.pain.0000000000000432, PMID: 26588696

[26] An JX He Y Qian XY Wu JP Xie YK Guo QL. A new animal model of trigeminal neuralgia produced by administration of cobra venom to the infraorbital nerve in the rat. Anesth Analg. (2011) 113:652–6. doi: 10.1213/ANE.0b013e3182245add, PMID: 21778333

[27] Zhu YL Xie ZL Wu YW Duan WR Xie YK. Early demyelination of primary A-fibers induces a rapid-onset of neuropathic pain in rat. Neuroscience. (2012) 200:186–98. doi: 10.1016/j.neuroscience.2011.10.037, PMID: 22061425

[28] Zhang L Song B Zhang X Jin M An L Han T. Resveratrol ameliorates trigeminal neuralgia-induced cognitive deficits by regulating neural ultrastructural Remodelling and the CREB/BDNF pathway in rats. Oxidative Med Cell Longev. (2022) 2022:4926678. doi: 10.1155/2022/4926678, PMID: 36478990 PMC9722315

[29] Ferdousi MI. Characterization of pain-, anxiety-, and cognition-related behaviors in the complete Freund's adjuvant model of chronic inflammatory pain in Wistar-Kyoto rats. Front Pain Res (Lausanne). (2023) 4:1131069. doi: 10.3389/fpain.2023.1131069, PMID: 37113211 PMC10126329

[30] Kondziolka D Lacomis D Niranjan A Mori Y Maesawa S Fellows W. Histological effects of trigeminal nerve radiosurgery in a primate model: implications for trigeminal neuralgia radiosurgery. Neurosurgery. (2000) 46:971–6. doi: 10.1097/00006123-200004000-00038 10764273

[31] Goldschmidt E Fellows-Mayle W Wolfe R Niranjan A Flickinger JC Lunsford LD. Radiosurgery to the spinal dorsal root ganglion induces fibrosis and inhibits satellite glial cell activation while preserving axonal neurotransmission. J Neurosurg Spine. (2020) 32:790–8. doi: 10.3171/2019.11.SPINE191176, PMID: 32005015

[32] Kiguchi N Kobayashi D Saika F Matsuzaki S Kishioka S. Inhibition of peripheral macrophages by nicotinic acetylcholine receptor agonists suppresses spinal microglial activation and neuropathic pain in mice with peripheral nerve injury. J Neuroinflammation. (2018) 15:96. doi: 10.1186/s12974-018-1133-5, PMID: 29587798 PMC5872578

[33] Samad TA Moore KA Sapirstein A Billet S Allchorne A Poole S. Interleukin-1beta-mediated induction of Cox-2 in the CNS contributes to inflammatory pain hypersensitivity. Nature. (2001) 410:471–5. doi: 10.1038/35068566, PMID: 11260714

[34] Liu MX. A correlative analysis between inflammatory cytokines and trigeminal neuralgia or hemifacial spasm. Neurol Res. (2019) 41:335–40. doi: 10.1080/01616412.2018.1564188, PMID: 30612530

[35] Patil S Testarelli L. Assessment of growth factors, cytokines, and cellular markers in saliva of patients with trigeminal neuralgia. Molecules. (2021) 26:10.3390/molecules26102964:964. doi: 10.3390/molecules26102964, PMID: 34067581 PMC8157075

[36] Yin Y. Pretreatment with resveratrol ameliorate trigeminal neuralgia by suppressing matrix metalloproteinase-9/2 in trigeminal ganglion. Int Immunopharmacol. (2019) 72:339–47. doi: 10.1016/j.intimp.2019.04.014, PMID: 31009895

[37] Boucher Y Moreau N Mauborgne A Dieb W. Lipopolysaccharide-mediated inflammatory priming potentiates painful post-traumatic trigeminal neuropathy. Physiol Behav. (2018) 194:497–504. doi: 10.1016/j.physbeh.2018.06.021, PMID: 29928887

[38] Lu J. Transplantation of olfactory ensheathing cells can alleviate neuroinflammatory responses in rats with trigeminal neuralgia. Brain Res. (2024) 1825:148732. doi: 10.1016/j.brainres.2023.148732, PMID: 38104922

[39] Lukacs M. KYNA analogue SZR72 modifies CFA-induced dural inflammation- regarding expression of pERK1/2 and IL-1beta in the rat trigeminal ganglion. J Headache Pain. (2016) 17:64. doi: 10.1186/s10194-016-0654-5, PMID: 27377707 PMC4932003

[40] Chen ML. NLRP3 inflammasome signaling as an early molecular response is negatively controlled by miR-186 in CFA-induced prosopalgia mice. Braz J Med Biol Res. (2018) 51:e7602. doi: 10.1590/1414-431X20187602, PMID: 30020320 PMC6050947

[41] Takeda M Kitagawa J Takahashi M Matsumoto S. Activation of interleukin-1beta receptor suppresses the voltage-gated potassium currents in the small-diameter trigeminal ganglion neurons following peripheral inflammation. Pain. (2008) 139:594–602. doi: 10.1016/j.pain.2008.06.015, PMID: 18694623

[42] Tanaka T Narazaki M Kishimoto T. IL-6 in inflammation, immunity, and disease. Cold Spring Harb Perspect Biol. (2014) 6:a016295. doi: 10.1101/cshperspect.a016295, PMID: 25190079 PMC4176007

[43] Khurshid Z Warsi I Moin SF Slowey PD Latif M Zohaib S. Biochemical analysis of oral fluids for disease detection. Adv Clin Chem. (2021) 100:205–53. doi: 10.1016/bs.acc.2020.04.005, PMID: 33453866

[44] He Z. The CSF1-CSF1R pathway in the trigeminal ganglion mediates trigeminal neuralgia via inflammatory responses in mice. Mol Biol Rep. (2024) 51:215. doi: 10.1007/s11033-023-09149-y, PMID: 38281257

[45] Latremoliere A Mauborgne A Masson J Bourgoin S Kayser V Hamon M. Differential implication of proinflammatory cytokine interleukin-6 in the development of cephalic versus extracephalic neuropathic pain in rats. J Neurosci. (2008) 28:8489–501. doi: 10.1523/JNEUROSCI.2552-08.2008, PMID: 18716207 PMC6671060

[46] Cambier S Gouwy M Proost P. The chemokines CXCL8 and CXCL12: molecular and functional properties, role in disease and efforts towards pharmacological intervention. Cell Mol Immunol. (2023) 20:217–51. doi: 10.1038/s41423-023-00974-6, PMID: 36725964 PMC9890491

[47] McGeachy MJ Cua DJ Gaffen SL. The IL-17 family of cytokines in health and disease. Immunity. (2019) 50:892–906. doi: 10.1016/j.immuni.2019.03.021, PMID: 30995505 PMC6474359

[48] Ihim SA Abubakar SD Zian Z Sasaki T Saffarioun M Maleknia S. Interleukin-18 cytokine in immunity, inflammation, and autoimmunity: biological role in induction, regulation, and treatment. Front Immunol. (2022) 13:919973. doi: 10.3389/fimmu.2022.919973, PMID: 36032110 PMC9410767

[49] Ostertag C. Heightened presence of inflammatory mediators in the cerebrospinal fluid of patients with trigeminal neuralgia. Pain Rep. (2023) 8:e1117. doi: 10.1097/PR9.0000000000001117, PMID: 38125050 PMC10732488

[50] van Loo G Bertrand MJM. Death by TNF: a road to inflammation. Nat Rev Immunol. (2023) 23:289–303. doi: 10.1038/s41577-022-00792-3, PMID: 36380021 PMC9665039

[51] Lis K Grygorowicz T Cudna A Szymkowski DE Balkowiec-Iskra E. Inhibition of TNF reduces mechanical orofacial hyperalgesia induced by complete Freund's adjuvant by a TRPV1-dependent mechanism in mice. Pharmacol Rep. (2017) 69:1380–5. doi: 10.1016/j.pharep.2017.05.013, PMID: 29132095

[52] Zeng Z Lan T Wei Y Wei X. CCL5/CCR5 axis in human diseases and related treatments. Genes Dis. (2022) 9:12–27. doi: 10.1016/j.gendis.2021.08.004, PMID: 34514075 PMC8423937

[53] Mori F Nistico R Nicoletti CG Zagaglia S Mandolesi G Piccinin S. RANTES correlates with inflammatory activity and synaptic excitability in multiple sclerosis. Mult Scler. (2016) 22:1405–12. doi: 10.1177/1352458515621796, PMID: 26733422

[54] Pittaluga A. CCL5-glutamate cross-talk in astrocyte-neuron communication in multiple sclerosis. Front Immunol. (2017) 8:1079. doi: 10.3389/fimmu.2017.01079, PMID: 28928746 PMC5591427

[55] Lechner J von Baehr V. Peripheral neuropathic facial/trigeminal pain and RANTES/CCL5 in jawbone cavitation. Evid Based Complement Alternat Med. (2015) 2015:582520. doi: 10.1155/2015/582520, PMID: 26170877 PMC4481083

[56] Gao YJ Zhang L Samad OA Suter MR Yasuhiko K Xu ZZ. JNK-induced MCP-1 production in spinal cord astrocytes contributes to central sensitization and neuropathic pain. J Neurosci. (2009) 29:4096–108. doi: 10.1523/JNEUROSCI.3623-08.2009, PMID: 19339605 PMC2682921

[57] Wei F. Supraspinal glial-neuronal interactions contribute to descending pain facilitation. J Neurosci. (2008) 28:10482–95. doi: 10.1523/JNEUROSCI.3593-08.2008, PMID: 18923025 PMC2660868

[58] Lu ZY. The up-regulation of TNF-α maintains trigeminal neuralgia by modulating MAPKs phosphorylation and BKCa channels in trigeminal nucleus Caudalis. Front Cell Neurosci. (2021) 15:764141. doi: 10.3389/fncel.2021.764141, PMID: 34899191 PMC8657151

[59] Dauvergne C Molet J Reaux-Le Goazigo A Mauborgne A Melik-Parsadaniantz S Boucher Y. Implication of the chemokine CCL2 in trigeminal nociception and traumatic neuropathic orofacial pain. Eur J Pain. (2014) 18:360–75. doi: 10.1002/j.1532-2149.2013.00377.x, PMID: 23918315

[60] Abu Hamdeh S Khoonsari PE Shevchenko G Gordh T Ericson H Kultima K. Increased CSF levels of apolipoproteins and complement factors in trigeminal neuralgia patients-in depth proteomic analysis using mass spectrometry. J Pain. (2020) 21:1075–84. doi: 10.1016/j.jpain.2020.03.002, PMID: 32553624

[61] Capelle HH Brandis A Tschan CA Krauss JK. Treatment of recurrent trigeminal neuralgia due to Teflon granuloma. J Headache Pain. (2010) 11:339–44. doi: 10.1007/s10194-010-0213-4, PMID: 20419329 PMC3476345

[62] Yang DB Jiang DY Chen HC Wang ZM. Second microvascular decompression for trigeminal neuralgia in recurrent cases after microvascular decompression. J Craniofac Surg. (2015) 26:491–4. doi: 10.1097/SCS.0000000000001523, PMID: 25759921

[63] Masuoka J Matsushima T Inoue K Nakahara Y Takase Y Kawashima M. Outcome of microvascular decompression for trigeminal neuralgia treated with the stitched sling retraction technique. Neurosurg Rev. (2015) 38:361–5. doi: 10.1007/s10143-015-0607-5, PMID: 25663308

[64] Steinberg JA Sack J Wilson B Weingarten D Carter B Khalessi A. Tentorial sling for microvascular decompression in patients with trigeminal neuralgia: a description of operative technique and clinical outcomes. J Neurosurg. (2018) 130:1315–20. doi: 10.3171/2017.10.JNS17971 29676696

[65] Ni H. Outcomes of treatment for elderly patients with trigeminal neuralgia: percutaneous balloon compression versus microvascular decompression. J Craniofac Surg. (2020) 31:e685-e688. doi: 10.1097/SCS.0000000000006544, PMID: 32472880

[66] Rapisarda A Baroni S Gentili V Moretti G Burattini B Sarlo F. The role of biomarkers in drug-resistant trigeminal neuralgia: a prospective study in patients submitted to surgical treatment. Neurol Sci. (2022) 43:4425–30. doi: 10.1007/s10072-022-05971-7, PMID: 35226213

[67] Sharouf F Hussain RN Hettipathirannahelage S Martin J Gray W Zaben M. C-reactive protein kinetics post elective cranial surgery. A prospective observational study. Br J Neurosurg. (2020) 34:46–50. doi: 10.1080/02688697.2019.1680795, PMID: 31645141

[68] Anderson LC Vakoula A Veinote R. Inflammatory hypersensitivity in a rat model of trigeminal neuropathic pain. Arch Oral Biol. (2003) 48:161–9. doi: 10.1016/s0003-9969(02)00203-0, PMID: 12642236

[69] Ma F Zhang L Lyons D Westlund KN. Orofacial neuropathic pain mouse model induced by trigeminal inflammatory compression (TIC) of the infraorbital nerve. Mol Brain. (2012) 5:44. doi: 10.1186/1756-6606-5-44, PMID: 23270529 PMC3563613

[70] Liu CY Lu ZY Li N Yu LH Zhao YF Ma B. The role of large-conductance, calcium-activated potassium channels in a rat model of trigeminal neuropathic pain. Cephalalgia. (2015) 35:16–35. doi: 10.1177/0333102414534083, PMID: 24820887

[71] Horie K Watanabe M Chanbora C Awada T Kunimatsu R Uchida T. Bovine lactoferrin reduces extra-territorial facial allodynia/hyperalgesia following a trigeminal nerve injury in the rat. Brain Res. (2017) 1669:89–96. doi: 10.1016/j.brainres.2017.04.015, PMID: 28465227

[72] Huang B Guo S Zhang Y Lin P Lin C Chen M. MiR-223-3p alleviates trigeminal neuropathic pain in the male mouse by targeting MKNK2 and MAPK/ERK signaling. Brain Behav. (2022) 12:e2634. doi: 10.1002/brb3.2634, PMID: 35608154 PMC9304854

[73] Bai X Zhang X Wang C Liu Y Liu X Fan Y. Stem cells from human exfoliated deciduous teeth attenuate trigeminal neuralgia in rats. Stem Cells Int. (2021) 2021:1–14. doi: 10.1155/2021/8819884, PMID: 33531911 PMC7834821

[74] Zhao Y Li T Zhang L Yang J Zhao F Wang Y. TRAF6 promotes spinal microglial M1 polarization to aggravate neuropathic pain by activating the c-JUN/NF-kB signaling pathway. Cell Biol Toxicol. (2024) 40:54. doi: 10.1007/s10565-024-09900-6, PMID: 38995476 PMC11245438

[75] Kingwell K. Pain: MAPK inhibitor shows promise in clinical trial for neuropathic pain. Nat Rev Neurol. (2011) 7:360. doi: 10.1038/nrneurol.2011.84, PMID: 21670759

[76] He Y Taylor N Fourgeaud L Bhattacharya A. The role of microglial P2X7: modulation of cell death and cytokine release. J Neuroinflammation. (2017) 14:135. doi: 10.1186/s12974-017-0904-8, PMID: 28716092 PMC5513370

[77] Liu H Ou C. Effect of lidocaine on expression of P2X7, p-p38 and IL-Iβ in the thalamus of rats with trigeminal neuralgia. Neurol Res. (2022) 44:1086–93. doi: 10.1080/01616412.2022.2112371, PMID: 36047574

[78] Bhattacharya A Lord B Grigoleit JS He Y Fraser I Campbell SN. Neuropsychopharmacology of JNJ-55308942: evaluation of a clinical candidate targeting P2X7 ion channels in animal models of neuroinflammation and anhedonia. Neuropsychopharmacology. (2018) 43:2586–96. doi: 10.1038/s41386-018-0141-6, PMID: 30026598 PMC6224414

[79] Recourt K van der Aart J Jacobs G de Kam M Drevets W van Nueten L. Characterisation of the pharmacodynamic effects of the P2X7 receptor antagonist JNJ-54175446 using an oral dexamphetamine challenge model in healthy males in a randomised, double-blind, placebo-controlled, multiple ascending dose trial. J Psychopharmacol. (2020) 34:1030–42. doi: 10.1177/0269881120914206, PMID: 32248747

[80] Dong B Xu R Lim M. The pathophysiology of trigeminal neuralgia: a molecular review. J Neurosurg. (2023) 139:1471–9. doi: 10.3171/2023.2.JNS23274, PMID: 37922556

